# Digital Technologies and the Role of Health Care Professionals: Scoping Review Exploring Nurses’ Skills in the Digital Era and in the Light of the COVID-19 Pandemic

**DOI:** 10.2196/37631

**Published:** 2022-10-04

**Authors:** Valentina Isidori, Francesco Diamanti, Lorenzo Gios, Giulia Malfatti, Francesca Perini, Andrea Nicolini, Jessica Longhini, Stefano Forti, Federica Fraschini, Giancarlo Bizzarri, Stefano Brancorsini, Alessandro Gaudino

**Affiliations:** 1 Department of Medicine and Surgery University of Perugia Terni Italy; 2 TrentinoSalute4.0 Centro di Competenza per la Sanità Digitale Trento Italy; 3 Azienda Provinciale per i Servizi Sanitari Trento Italy; 4 Azienda Unità Sanitaria Locale Umbria 2 Terni Italy; 5 Amministratore Unico di PuntoZero Perugia Italy

**Keywords:** role of the nurse in telemedicine, telenursing, new technological approaches, communication and technological skills, leadership, nursing training, nursing, health care, digital knowledge, online health, digital health, COVID-19, telehealth, telemedicine

## Abstract

**Background:**

The nursing role significantly changed following reforms in the nurse training process. Nowadays, nurses are increasingly trained to promote and improve the quality of clinical practice and to provide support in the assistance of patients and communities. Opportunities and threats are emerging as a consequence of the introduction of new disruptive technologies in public health, which requires the health care staff to develop new digital skills.

**Objective:**

The aim of this paper is to review and define the role of nurses and the skills they are asked to master in terms of new methodological approaches and digital knowledge in a continuously evolving health care scenario that relies increasingly more on technology and digital solutions.

**Methods:**

This scoping review was conducted using a thematic summary of previous studies. Authors collected publications through a cross-database search (PubMed, Web of Science, Google Scholar) related to new telemedicine approaches impacting the nurses’ role, considering the time span of 2011-2021 and therefore including experiences and publications related to the first phase of the COVID-19 pandemic.

**Results:**

The assessment was completed between April and July 2021. After a cross-database search, authors reviewed a selection of 60 studies. The results obtained were organized into 5 emerging macro areas: (1) leadership (nurses are expected to show leadership capabilities when introducing new technologies in health care practices, considering their pivotal role in coordinating various professional figures and the patient), (2) soft skills (new communication skills, adaptiveness, and problem solving are needed to adapt the interaction to the level of digital skills and digital knowledge of the patient), (3) training (specific subjects need to be added to nursing training to boost the adoption of new communication and technological skills, enabling health care professionals to largely and effectively use new digital tools), (4) remote management of COVID-19 or chronic patients during the pandemic (a role that has proved to be fundamental is the community and family nurse and health care systems are adopting novel assistance models to support patients at home and to enable decentralization of services from hospitals to the territory), and (5) management of interpersonal relationships with patients through telemedicine (a person-centered approach with an open and sensitive attitude seems to be even more important in the framework of telemedicine where a face-to-face session is not possible and therefore nonverbal indicators are more problematic to be noticed).

**Conclusions:**

Further advancing nurses’ readiness in adopting telemedicine requires an integrated approach, including combination of technical knowledge, management abilities, soft skills, and communication skills. This scoping review provides a wide-ranging and general—albeit valuable—starting point to identify these core competences and better understand their implications in terms of present and future health care professionals’ roles.

## Introduction

Over the years, the nursing profession has been exposed to relevant changes, from the mother-rescuer’s role to the intuitions of Florence Nightingale [1,2] and considering the reforms in the nurse training process, which now requires a 3-year university degree in most countries [2,3].

Thanks to higher education curricula and advanced educational training, nurses have become increasingly prepared, competent, and autonomous [1,3]. Even if this advancement has led to a tangible improvement in the quality of clinical practice and assistance to the person and the community [4], much more has to be done considering the wide-ranging digital health revolution we are witnessing over the past years. Nowadays, health care is undergoing profound changes, moving toward the decentralization of services and promoting outpatient activities. The delivery of health care treatments with the support of new technologies has an increasingly significant impact on the health care management and organizational asset.

This new scenario highlights the need for highly skilled nursing staff, able to adapt to new contexts and challenges that are constantly and quickly emerging within the digital health revolution [5].

In this rapidly changing context, the training of nursing skills and competences need to include the use and administration of new technologies, as well as the capacity to support the use of technological tools for patients and caregivers [6], identifying those who are eligible candidates for services enabled by digital technologies [7].

The whole process has been inevitably accelerated by the COVID-19 pandemic, which is highlighting the limited amount of resources in terms of health care staff and in terms of technological tools. The pandemic is also pushing health care institutions in reshaping service delivery, the management of patients’ journeys, and the use of telemedicine [8].

The aim of this paper is to review and define the role of nurses and the skills they are asked to master in terms of new methodological approaches and digital knowledge, considering the changes that have occurred in the health sector in the past decade (2011-2021). The COVID-19 pandemic has led to an exponential increase in the use of telemedicine, and consequently, this has led to a radical change in the health care organization and in the role of professionals as well, particularly in the past couple of years. In fact, a large part of the available literature (including reviews) is related to a pre–COVID-19 period. Despite a considerable number of current reviews including robust systematic and statistical analysis, these papers (1) mainly address the issue of telemedicine only as case studies or isolated experiments, focusing on the perception of its use among nurses and students and on possible future prospects, underlining the main limitations [9,10], and (2) somehow miss detecting the massive impact of the pandemic on the health care sector and its recent acceleration in terms of the digital revolution.

In the continuum of experiences that have occurred in the past 10 years in the field of digitalization of the health care sector, this review also covers the recent COVID-19 outbreak that has contributed to largely expand telemedicine across several countries, with the aim of mapping the key concepts and implications underpinning the fast/changing role of the nurse in the digital era, as well as exploring the boundaries of the role and the skills that need to be acquired as they are redefined through the COVID-19 pandemic.

Therefore, this paper can be considered a preliminary—albeit necessary—exercise prior to conducting a structured systematic review.

## Methods

### Aim

The core objective is to review and define the role of nurses and the skills they are asked to master in terms of new methodological approaches and digital knowledge that have emerged before and during the COVID-19 pandemic (2011-2021). The review maps present (and future) skills that nurses are expected to develop when using new technologies, considering the novel organizational assets that are emerging in the digitalization process of health care.

### Design

From a methodological viewpoint, the scoping review has been identified as the most suitable tool as this approach is particularly useful for exploring new evidence, embracing evidence from heterogeneous sources of data, and providing a broad overview of the current and rapidly changing health care scenario [11]. Authors performed a scoping review [12-14]. In line with the scoping review principles, the steps of the review were as follows: (1) defining the focus of the review, (2) identifying relevant studies using inclusion and exclusion criteria, (3) charting the studies, and (4) summarizing the core results. Results were analyzed adopting a thematic synthesis approach. Further details about the method adopted will be presented in the following sections.

### Search Strategy

The search strategy was defined as follows:

- Participants: nursing professionals, researchers, and university professors
- Interest: application of telehealth in nursing practice
- International electronic databases: PubMed, Google Scholar, and Web of Science, considering the time span of 2011-2021
- Keywords used: “telenursing,” “nursing,” and “telemedicine”
- Search strings: (telenursing) AND (role of the nurse in telemedicine), (nurse's role) AND (eHealth), (nurse's role) AND (telemedicine COVID-19), ((community nurse) AND (telemedicine)) AND (COVID-19), and (telenursing) AND (telemedicine)

Authors conducted the research through the Perugia University library (Italy) using PubMed, Google Scholar, and Web of Science. The search was planned considering a 10-year span from 2011 to 2021.

#### Inclusion and Exclusion Criteria

Papers from 2011 to 2021 were identified using the keyword search strings through the PICO (population, intervention, control, outcome) method. During screening, all papers meeting the following selection criteria were selected: written in English; being of international significance; being searched through a cross-database search (PubMed, Web of Science, Google Scholar), considering elimination of redundant papers; including a reference to the nursing figure and the role of the nurse, with reference to the leadership role; and including the use of telemedicine systems. In addition, papers considering at least 1 of the following issues/topics were selected: nursing education and introducing clinical practice supported by telemedicine systems into the curriculum, interpersonal and communication skills in terms of telemedicine systems, and telemedicine experience (see Table 1). The final list included 60 papers.

**Table 1:** Paper selection process.

Step	Process
Identification	In total, 250 papers written in English, with international significance, in the time span of 2011-2021 were identified through the PICO method based on the keywords selected: (telenursing) AND (role of the nurse in telemedicine), (nurse's role) AND (eHealth), (nurse's role) AND (telemedicine COVID-19), ((community nurse) AND (telemedicine)) AND (COVID-19), and (telenursing) AND (telemedicine). No additional records were found from other searches.
Screening	A cross-database search (PubMed, Web of Science, Google Scholar) allowed the elimination of 8 (3.2%) redundant papers. The remaining 242 (96.8%) papers were reviewed, and 156 (64.5%) that did not have free full text and did not meet the purpose of the research were excluded.
Eligibility	All the remaining 86 (35.5%) papers with free full text were considered eligible. Of these, 26 (30.2%) papers with redundant topics not in sufficient detail were further excluded.
Included	Finally, 60 (69.8%) papers were included.

#### Publication Assessment

An assessment of the risk of bias in the publications is usually not performed in the framework of a scoping review; therefore, no assessments of the papers' quality were performed [14].

### Data Abstraction and Synthesis

After collection, the papers were divided and analyzed. The results of authors’ analysis are presented in Tables A1-A3 in Multimedia Appendix 1. The tables summarize the core pieces of information for each item: numerical code, title of the paper, aim, journal, authors, year, telehealth/eHealth application, data collection methods, and summary of contents.

## Results

### Search Outcomes

**Figure 1:**
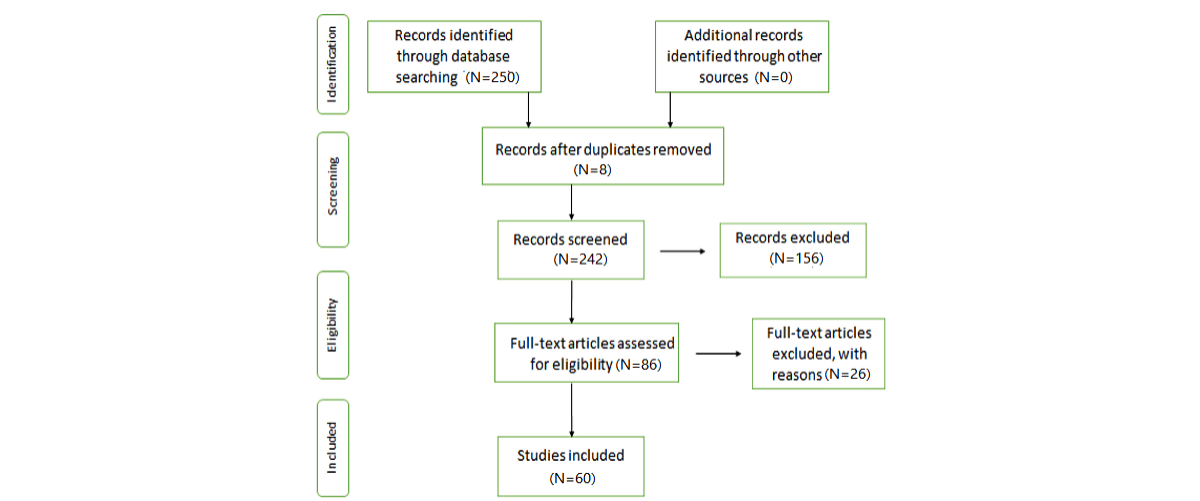
Preferred Reporting Items for Systematic Reviews and Meta-Analysis (PRISMA) flowchart for the scoping review process.

In the initial phase, authors selected many papers from international peer-reviewed journals. In total, 250 papers were found matching the topic of the review. After reading the abstracts, papers that were not in line with the inclusion criteria of the review were removed. A total of 60 (24%) papers were selected to be reviewed. Of these 60 papers, 22 (37%) covered the period 2011-2018 and were therefore not affected by the COVID-19 pandemic (process summarized in the Preferred Reporting Items for Systematic Reviews and Meta-Analysis (PRISMA) flowchart in Figure 1).

Specific recurring themes and fundamental constructs that emerged in this review are presented in the following sections and then elaborated in the Discussion section.

### Communication Skills in Telemedicine for Nurses

This scoping review highlighted the importance of communication skills that appear to be even more important for nurses in the case of telemedicine compared to in-person visits [15]. Communication abilities and the capacity to manage interpersonal relations with patients through telemedicine tools are described as key abilities. In this case, solid professional experience is evidenced to be a vital factor in ensuring ideal quality in organizational assets supported by new technologies. This research explored the importance of improvements telemedicine systems and informatics in nurse practitioner (NP) education [5,16]. Recent research from the American Academy of Family Physicians (AAFP) highlighted the lack of training as 1 of the most common barriers to using telehealth [7]. Furthermore, another systematic review emphasized insufficient access and lack of training and education as key factors in the nonadoption of new technologies [7]. For these reasons, international organizations (eg, the Commission on Accreditation for Health Informatics and Information Management Education and the International Medical Informatics Association) have proposed a new mobile health (mHealth) skills training framework to prepare health professionals to deploy and implement mHealth interventions. This framework considers teaching digital communication skills, technology literacy and usage skills, telehealth business cases, regulatory and compliance issues, interprofessional teams, and deploying of telehealth products and services [7]. The skills are considered as basic competences to conduct remote consultation, monitor, diagnose, and treat patients. The final goal is to expand the use of digital tools and technological devices in different health care contexts, such as telepresence robots, patient-monitoring devices, pulse oximeters, blood pressure monitors, radiofrequency identification and temperature sensors, electrocardiography (ECG), and others.

From this perspective, the following list [7] of skills emerged as core competencies:

- Technical skills to use telemedicine (system use and troubleshooting telemedicine software and hardware issues)
- Skills in terms of using mobile communication devices and wireless remote patient-monitoring applications for treatment support, chronic disease management, and disease surveillance
- Skills in designing patient-centered health informatics systems
- Skills in understanding specific health informatics issues and suggesting/designing technological solutions
- Skills to assess data integrity and health data analysis

It is therefore possible to affirm that there is a need to recommend specific skill training; however, a clear and effective training method to properly train clinicians is far from being determined yet. The current education based on traditional lectured instruction methods is presumably not sufficient for proper adoption of new technologies, leading to bottlenecks in integrating monitoring technologies into daily practice.

### Management of Interpersonal Relationships With Patients Through Telemedicine

Telehealth nursing primarily focuses on patients’ long-term wellness, self-management, and health. According to the American Telemedicine Association, information technology (IT) solutions provide the delivery of nursing care, regardless of distance, while expanding the care providers’ ability to monitor, educate, follow up, collect data, and provide multidisciplinary care, including remote interventions, pain management, and family support, in an innovative fashion [17]. When targeting elderly people, 1 of the emerging skills for nurses adopting telemedicine is communication, which is essential in telephone counseling. A person-centered approach with an open and sensitive attitude seems to be even more important in the framework of telemedicine where a face-to-face (FTF) session is not possible and therefore nonverbal indicators are more problematic to be noticed.

Another relevant item to consider is the nurses' working environment, which should be carefully considered. A quiet, disturbance-free environment was clearly indicated as a core issue when delivering telemedicine sessions in order to promote a contextual environment supporting mutual understanding and smooth interaction [18].

### Remote Management of COVID-19 or Chronic Patients During the Pandemic

A role that has proved to be fundamental is the community and family nurse. Health care systems are adopting novel assistance models to support patients at home and to enable decentralization of services from hospitals to the territory [19]. The process was further fostered—and it is still evolving—by the recent COVID-19 pandemic, which represents an engine of massive acceleration for digital health. During the strict lockdown period, the reduction of routine FTF care, the provisional interruption of most outpatient care services and the stay-at-home directives added a significant pressure to the community component of health care systems.

From this perspective, community nurses play a key role in integrating health and social care services, particularly supporting self-care that has also proved to be beneficial during the pandemic. Community nurses have shifted from FTF care to teleconsultations to keep services accessible during the pandemic [19]. If this was possible for standard support, during the most critical phases of the COVID-19 pandemic, a reduced level of direct assistance was experienced from patients in the last stage of the disease, given the need for isolation at home. To overcome this problem, in some areas, end-of-life health care services are delivered remotely and support to the families/patients can be managed through technological platforms [20]. UK national guidelines have been delivered to allow family caregivers to administer end-of-life medications, if appropriate, and have access to timely clinical advice. Often, district nursing teams provide ongoing training and support to family caregivers willing to take on increased responsibility for patient care and associated management techniques. One of the enduring implications of the COVID-19 pandemic is also the increased need to deliver remote consultations in the field of end-of-life planning and palliative care [20].

eHealth can be supported by rapid counseling models based on remote calling by health care professionals, as shown in a randomized controlled trial [21] that evaluated the effect of remote nursing-monitoring of overweight women and confirmed the benefits in terms of improved anthropometric measurements.

This study, therefore, allows us to understand the positive effect of this approach (monitoring via phone calls) and confirms the effectiveness of the home remote control method, mainly as an educational strategy, for flexibility of schedules, optimization of times and resources, and the ability to reach many users who encounter difficulties related to geographical and financial barriers to access the health service.

### Management and Leadership Within Advanced Telemedicine Systems

The nurse is increasingly becoming a key figure for the implementation of telemedicine in clinical practice. This requires specific leadership skills. Leaders set standards, develop plans, and remove barriers to implementation. The leader understands and can deliver and adapt evidence-based practice knowledge. The leader supports the efforts of others to learn and use evidence-based practice, as well as supports the implementation of evidence-based practice in a consistent and deliberate way [22]. The nurse plays a fundamental role in assistance supported by telemedicine systems. In fact, as demonstrated by Parimbelli et al [6], the nurse is the figure who participates in the creation of the project and is responsible for the patient’s enrollment phase, for the explanation of the service to the patient and the caregiver, and, finally, for the management of the data they receive through the device used. As modes of delivery for services such as telehealth and telenursing changes, nurses are increasingly working independently and using information and communication technologies to collaborate with the health team. The goals for the future are better use of technology and information, promotion and dissemination of innovation and quality improvements, and the creation of leaders and capacities in terms of administering new technologies. This means that nursing care must focus on the delivery of services such as telemedicine and teleassistance [23].

### Impact of Nurses’ Previous Professional Experience When Using Telemedicine

If the technology is not optimal, more experienced nurses can compensate the suboptimal technology with their clinical experience, while younger nurses often face bigger challenges, despite their higher digital skills. In some cases, it is emphasized that digital natives often have more doubts about the correct application of digital tools in health care. The study titled “Telehealth and Telenursing Perception and Knowledge Among University Students of Nursing in Poland” [24] was conducted to investigate the perception of telenursing among nursing students, providing some predictive data for the near-future nursing practice in the country. The current generation of nursing students appear to be well qualified in the use of IT and medical technology (personal computers, personal email, etc). They seem to be better prepared for joining the information society, including the practice of telemedicine. Most of the students interviewed reported a positive attitude toward the use of telenursing. The fact that a significant number of students are familiar with the terms “telemedicine” and “telenursing” could be a prognostic factor in the future development of telenursing [24].

## Discussion

### Principal Findings

This review aimed at exploring the present (and future) nursing role, considering the new digital health era where the use of telemedicine is becoming a standard in health care delivery. From a methodological viewpoint, the scoping review represents the most suitable tool for this exercise, as this approach is appropriate when exploring emerging evidence from heterogeneous sources of data [25,26].

Revising the literature published within a 10-year span, authors were able to outline (1) the emerging skills and attitudes nurses are required to master within a digitalized health care system, (2) the new responsibilities that are emerging for health care staff (and nurses in particular) when digital health approaches are adopted, and (3) the (potential) impact of digital innovation in terms of requirements for the nurses’ university curricula.

Among the core emerging issues highlighted in this paper, the leadership role that the nurse plays is key when new technologies are structurally part of the health care process and service delivery. In fact, nurses are (and will increasingly be) the cornerstone of communication and coordination among different professional stakeholders (eg, general practitioners, specialists), patients, and caregivers. Another important point emerging from our analysis is the need for developing new communication, adaptation, and problem-solving capabilities to flexibly adapt the use of technologies to the level of digital literacy (digital skills) of patients. This implies the capacity to assess not only the clinical profile of a patient but also the extent to which new technologies can be used in managing a specific case. This is even more important, considering an increasing body of publications [16,24] highlighting the key role of telemedicine also during first contacts with patients, in the case of urban, rural, and remote settings. This is the case for developing countries but also in industrialized areas (eg, Canada) and in emergency scenarios (eg, pandemic). Finally, the need for introducing transversal, communicative, and technological skills in the university curricula of nurses was highlighted in order to improve their capability of using and adapting new technological approaches to current (and future) health care delivery pathways.

### Limitations

The authors acknowledge some limitations of this review. First, the level of eHealth literacy of nurses was assessed only in a limited number of papers, in many cases outdated, as in the case of Norman and colleagues [25,26]. This can be considered an intrinsic limitation for this kind of study. Second, potentially relevant sources of information may have been omitted because of different wording that is sometimes adopted in the literature targeting telemedicine. In addition, the wording adopted for the review might have led to potential biases. In addition, the study considered health care approaches supported by telemedicine systems as its main topic, which is constantly evolving, especially after the COVID-19 pandemic. Future studies can further explore the multifaced impact of the COVID-19 pandemic on the health care system. Another limitation is the scarce presence of papers relating to experiences in the field of nursing care supported by telemedicine systems. The nursing role in terms of new technologies is not yet well defined; in fact, this study can represent a basis from which to start to define a nurse with new specific skills and technological capabilities. Only when these skills are included in the educational path will it be possible to witness the detailed delineation of the nurse of the future in terms of technological knowledge. Finally, a core limitation lies in the lack of a standard curriculum throughout Europe regarding the nursing figure. This does not allow a clear mapping of proper strategies to integrate technological skills into educational courses for nurses.

### Conclusion

As highlighted in this review, the role of nurses has widely changed over the past decades, with tremendous acceleration in the past few years and in the COVID-19 era. Studies included in this scoping review provide insight into present and future skills that might constitute the core abilities of the nurses’ curricula. At the same time, additional information is needed to better understand how these skills can be included in the training and nurses’ formal curricula, particularly considering the radical transformation we are witnessing, where the traditional models of care are continuously modified and sometimes replaced with advanced approaches enabled by up-to-date technologies. Our research can be considered a starting point to outline the core areas of development for the future nurse, in terms of technological, organizational, and relational skills [27]. These areas of development clearly impact the training curricula that are still wide ranging at a global level, leading to a still fragmented scenario in terms of digitally skilled nurses. This lack of professional nurses familiar with telemedicine is recurrent evidence across the literature [8,16]. The large majority of the selected papers refer to experiments or possible future training initiatives that are not yet in place, calling for a revolution also in terms of educational curricula in order to further improve the capacity and flexibility of nurses in the digital era. This issue needs to be further investigated through future research.

## Appendix

Multimedia Appendix 1 Results of authors’ analysis of 60 papers selected for this study.

## Abbreviations

ECG: electrocardiography
FTF: face-to-face
IT: information technology
mHealth: mobile health
NP: nurse practitioner
PICO: population, intervention, control, outcome
