# Supplementary material for: Digital Technologies and the Role of Health Care Professionals: Scoping Review Exploring Nurses’ Skills in the Digital Era and in the Light of the COVID-19 Pandemic
Source: JMIR Nurs. 2022 Oct 4;5(1):e37631. doi: 10.2196/37631 (PMC9579937; doi:10.2196/37631)
Supplement: Multimedia Appendix 1 [file nursing_v5i1e37631_app1.docx]

**Table A1.** Summary of papers 1-20.

| Study number | Title | Aim | Journal/publisher; authors (year) | Telehealth/eHealth application | Data collection method | Key conclusion |  |
| --- | --- | --- | --- | --- | --- | --- | --- |
| 1 | The Role of Telehealth During the COVID-19 Pandemic Across the Interdisciplinary Cancer Team: Implications for Practice | Role of telemedicine during the COVID-19 pandemic for cancer patients by a multidisciplinary team | Elsevier; Paterson et al (2020) | Remote monitoring | Literature review | - Oncology nurses can make a central contribution to the delivery of telemedicine through transformational leadership across all domains and settings in cancer care. - Telemedicine provides a solution to the current global health crisis, but it could also benefit the future provision of large-scale clinical trials and services. |  |
| 2 | Nursing Professionals' Experiences of the Facilitators and Barriers to the Use of Telehealth Applications: A Systematic Review of Qualitative Studies | Evaluating the pros and cons of telemedicine used by nurses and how to allow the implementation of the various tools in daily nursing care | Wiley Blackwell; Koivune and Sarato (2018) | Remote monitoring | Systematic review of qualitative studies | - New technologies have not led to major changes over the years among nurses. - The advancement in telemedicine will lead nurses to increase their skills in this new area, pushing the patients they follow into this new world as well. | |
| 3 | Sensor Technology for Nursing Research | The use of new sensors for measuring vital parameters, even remotely | *Nursing Outlook*; Redeker (2020) | Sensors for monitoring vital signs | Literature review | N/A^a^ | |
| 4 | eHealth Systems for the Optimised Care of Patients With Type 2 Diabetes | Remote monitoring of patients with type 2 diabetes becoming a common nursing practice; greater implementation of these skills | *British Journal of Nursing*; Klösch et al (2020) | eHealth methods | Literature review | - In the future, increasingly more nurses will need to integrate eHealth systems into the treatment of patients with type 2 diabetes. - It is important that nurses understand these systems to ensure successful and sustainable implementation. | |
| 5 | Perspectives of Nurses Toward Telehealth Efficacy and Quality of Health Care: Pilot Study | Evaluating daily remote monitoring of chronic patients managed by remote nurses | JMIR Publications; Bashir and Bastola (2018) | SERVUQUAL instrument and telehealth | Pilot study | - The study highlighted how the telemedicine process provides daily patient health monitoring, leading to the benefits of immediate feedback for patients, family, and health care professionals, as well as the convenience of scheduling. | |
| 6 | Photography in Telemedicine: Improving Diagnosis of Chronic Graft-Versus-Host Disease | Improving the detection and management of graft-versus-host disease through telemedicine with educational tools designed in collaboration with nursing students | *Clinical Journal of Oncology Nursing*; Busby (2017) | Brochure and video | Experiment | N/A | |
| 7 | Telemedicine and Telehealth in Nursing Homes: An Integrative Review | Telemedicine capable of reducing the risk of complications by avoiding inappropriate or preventable hospital access | *Journal of the American Medical Directors Association*; Groom et al (2021) | Telemedicine and telehealth | Integrative review | - Measurable impacts, such as reduced emergency and hospital admissions, financial savings, reduced physical restrictions, and improved vital signs, were found along with process improvements, such as convenient access to specialists. | |
| 8 | The Nurse LEADership for Implementing Technologies: Mobile Health Model | Skills and knowledge that Canadian nurses must have for the implementation of technology in their work | Longwoods; Ronquillo et al (2019) | mHealth technologies | Conceptual models | - The Nurse LEAD-IT model can help outline the skills and knowledge needed by nursing leaders to successfully implement mHealth initiatives in nursing practice. | |
| 9 | Digital Continuous Health Care and Disruptive Medical Technologies: m-Health and Telemedicine Skills Training for Data-Driven Health Care | Study carried out on 206 nursing students to evaluate their skills in the use of telemedicine, many wanting to deepen the subject to increase their confidence with wearable and nonwearable technology | SAGE journals; Sapci and Sapci (2019) | Wireless monitoring devices | Interviews, observation, and analyses of archival data from nurses’ records | - Propose a skills training framework covering telemedicine, mHealth and connected health, health care IT^b^ application development. and health care IT device innovation. | |
| 10 | Telenursing in Incidents and Disasters: A Systematic Review of the Literature | Evaluating the functionality of telemedicine and telenursing in an emergency context so as to allow an improvement in the assistance response | *Journal of Emergency Nursing* (2020) | Telenursing methods | Systematic review | - Telenursing is important to try to reduce the inconvenience due to emergencies and disasters, providing a new window for assistance. | |
| 11 | Methodologies for Improving the Quality and Safety of Telehealth Systems | Telemedicine useful for supporting Canadian rural areas through 2 simulations, 1 based on the application of teletriage and 1 on telenursing | IOS Press; Borycki and Kushniruk (2019) | Simulation for telehealth applications | Experiment | N/A | |
| 12 | Prevalence of Telehealth in Nursing: Implications for Regulation and Education in the Era of Value-Based Care | To demonstrate the need for new and renewed skills to provide secure, effective, and culturally relevant telematic and virtual assistance | SAGE journals; Rambur et al (2019) | Telehealth and telephone calls | N/A | N/A | |
| 13 | Assessing the Impact of Telehealth Objective Structured Clinical Examinations in Graduate Nursing Education | To describe how the implementation of teleassistance simulations in the preparation of nursing students can increase skills and benefits in the future profession | *Nurse Educator*; Phillips et al (2020) | No-cost telehealth simulation | N/A | - The use of telemedicine simulation can be an effective strategy for assessing clinical competence, providing personalized feedback, and ensuring that students use evidence-based practice. | |
| 14 | The Role of Nurses in E-Health: The MobiGuide Project Experience | Role of the nurse in developing, distributing, and evaluating the usefulness of eHealth, nurses being responsible for the enrollment and training of patients, daily data check, and the final phase of the study where patients are interviewed about their experience with the system | IOS Press; Parimbelli et al (2016) | Home monitoring with ECG^c^ sensor and telephone | Project experience | N/A | |
| 15 | Telemonitoring by the Nurse of Patients Equipped With an Implantable Heart Device | Telemedicine training for nurses to provide them with basic skills to deal with the care of decompensated patients through remote rhythmology | Elsevier; Guédon-Moreau et al (2016) | Remote monitoring through implantable device | Project experience | N/A | |
| 16 | Caring From a Distance: The Role of Telehealth | To understand the barriers preventing telemedicine from being implemented in nursing practice, including providing good instruction | Ovid; Taylor and Coates (2015) | Telehealth and case studies | Quantitative study through case studies | - Many of the barriers to using telemedicine can be avoided with better planning and collaboration | |
| 17 | Examining the Use of Telehealth in Community Nursing: Identifying the Factors Affecting Frontline Staff Acceptance and Telehealth Adoption | To understand the degree of acceptance of telemedicine by nurses in the monitoring of patients with obstructive pulmonary and cardiac problems | Wiley Blackwell; Taylor et al (2015) | Case studies about telehealth | Quantitative review | - Doctors must be able to tackle the barriers of telemedicine if they are to succeed in adopting it in daily practice. They cannot afford to lose confidence at the first difficulties. | |
| 18 | Telehealth Simulation With Graduate Nurse Practitioner Students | Simulation carried out to understand the degree of preparation of nurses regarding the use of telemedicine, with assessments before and after the practical test | *Nurse Educator*; Emerson et al (2021) | Telehealth simulation training experience | Quantitative study | - This telemedicine simulation training experience provided NP^d^ students with an opportunity to engage in activities to enhance their knowledge and preparation in providing assistance through telemedicine in a rural setting. | |
| 19 | Effects of Telehealth by Allied Health Professionals and Nurses in Rural and Remote Areas: A Systematic Review and Meta-Analysis | Importance of telemedicine in rural areas to provide services on par with those performed in person by health professionals, thus reducing costs | *Journal of Rehabilitation Medicine*; Speyer (2018) | Case studies before and after | Systematic review and meta-analysis | - Telemedicine services can be as effective as FTF^e^ interventions, which is encouraging, given the potential benefits of telemedicine in rural and remote areas in terms of access to health care and time and cost savings. | |
| 20 | Enhancing Telehealth Education in Nursing: Applying King's Conceptual Framework and Theory of Goal Attainment | In a university nursing center, attempting to implement telemedicine as a subject of study using King's conceptual system and the theory of achievement of objectives as a guide to training | SAGE journals; Fronczek et al (2017) | Telehealth through King’s systems-based approach | Experience | - Although technology is an increasingly essential element of health care, it should not replace the unique nurse-patient relationship. From an educational point of view, faculty must also ensure that they provide opportunities for nursing students to blend the art of nursing with the science of technology. | |

^a^N/A: not applicable.

^b^IT: information technology.

^c^ECG: electrocardiography.

^d^NP: nurse practitioner.

^e^FTF: face-to-face.

**Table A2.** Summary of papers 21-40.

| Study number | Title | Aim | Journal/publisher; authors (year) | Telehealth/eHealth application | Data collection method | Key conclusion |
| --- | --- | --- | --- | --- | --- | --- |
| 21 | Telehealth in Psychiatric Nursing Education: Lessons From the Field | Telepsychiatry for the training of nurses who are pursuing a master's degree in psychiatry and mental health through online internships | *Journal of the American Psychiatric Nurses Association*; Tyson et al (2019) | Telepsychiatry | Experience | - Despite the importance of technology in assistance, this must not replace the figure of the psychiatric nurse but must support them in their work. - Telepsychiatry is important as a means of education and for carrying out internships. |
| 22 | Telehealth Alerts and Nurse Response | Review of 187 medical records of elderly patients assisted in the area through telemedicine and telemonitoring systems based on alert systems managed directly by nurses | Mary Ann Liebert Inc; Murphy (2018) | Telehealth alert | Quantitative review | - In the future, it will be necessary to try to reduce the alerts that did not arise from clinical worsening, with the possibility of recognizing the progress of the disease by both the patient and the nurse. |
| 23 | Nursing Care by Telehealth: What Is the Influence of Distance on Communication? | To understand the pros and cons of telemedicine through a questionnaire | SciELO; Almeida Barbosa (2017) | Telehealth | Qualitative review | - The perception of nurses working in telemedicine in Brazil is that technology has facilitated their professional practice; however, in relation to the communication process, they believe that it is more difficult to communicate by telemedicine, mainly due to the difficulty in perceiving nonverbal signals (human part is missing). To overcome these difficulties, they agreed that interpersonal communication is a skill that must be acquired during their professional training over time. |
| 24 | Rise of the e-Nurse: The Power of Social Media in Nursing | Paper relating to the usefulness of nursing education regarding electronic literacy for an increasingly aware use of technologies in assistance | *Contemporary Nurse*; Ross and Cross (2019) | Nursing education | N/A^a^ | - Nursing education must interact with technology and social media to provide a future-ready workforce capable of delivering future eHealth reforms. |
| 25 | Community Nursing Services During the COVID-19 Pandemic: The Singapore Experience | Experience of community nurses in Singapore, who reacted to the COVID-19 crisis through telemedicine systems for the assistance of vulnerable elderly people with chronic health conditions | *British Journal of Community Nursing*; Yi et al (2020) | Teleconsultation | Report | - The goal is to share the Singapore General Hospital community nursing experience, preparedness, and transformation efforts during the pandemic. The experience gained has been invaluable in advancing future community nursing services in the evolving health care landscape. - Structured teleconsultation and technological advancement are useful for expanding the quality of community nursing services in the evolving health care landscape. |
| 26 | Enhancing Communication Skills for Telehealth: Development and Implementation of a Teach-Back Intervention for a National Maternal and Child Health Helpline in Australia | Experience related to the communication skills of telenurses, through the teaching of the Teach-Back technique, used in a national telephone helpline for maternal and child health | *BMC*; Morony et al (2018) | Communication skills for telehealth | Qualitative study | - Experience has made nurses favorable to the use of Teach-Back as a communicative methodology, useful in telemedicine systems and therefore to be included in the training course of the telenurse. |
| 27 | Rethinking Presence: A Grounded Theory of Nurses and Teleconsultation | Video teaching for nurses on the importance of using telenursing in clinical practice | *Journal of Clinical Nursing*; Barret (2017) | Semistructured interviews | Straussian analytical approaches | - This study provides nurses with information about the impact of teleconsulting on their professional role and an understanding of how to best use video-mediated communication to support patient care. |
| 28 | Advocacy of Home Telehealth Care Among Consumers With Chronic Conditions | Description of the use of home telematic assistance as an alternative for the management of chronic diseases from the users' point of view. | *Journal of Clinical Nursing*; Lu et al (2014) | Home telehealth care | Qualitative study | - To support home telematic care, nurses must play an active role in providing consumers with adequate training and support for any problem when they adopt the system to foster patient readiness to use this service. |
| 29 | Evolving National Strategy Driving Nursing Informatics in New Zealand | Three areas of health strategy in New Zealand: the best use of technology and information, the promotion and dissemination of innovation and quality improvements, and building leaders and capabilities for the future | IOS Press; Honey and Westbrooke (2016) | Telemedicine and telenursing | N/A | - Nurses are increasingly working independently and using information and communication technologies to collaborate with the health care team. |
| 30 | It Is Important That They Care: Older Persons' Experiences of Telephone Advice Nursing | To explore seniors' experiences of nursing telephone counseling at primary health care centers | *Journal of Clinical Nursing*; Holmström et al (2016) | Telenursing | Qualitative study | - This study contributes to a greater awareness of the advantages and disadvantages of the telephone counseling nursing system experienced by older people. |
| 31 | A Systematic Review of Nurses' Perspectives Toward the Telemedicine Intensive Care Unit: A Basis for Supporting Its Future Implementation in China? | Critique and summary of existing research on the perspectives of intensive care unit (ICU) nurses toward the telemedicine intensive care unit (tele-ICU), exploring the evidence base to support potential future implementation of the tele-ICU program in China | Mary Ann Liebert Inc; Li and Cotton (2019) | Telemedicine in the ICU | Systematic review | - This review summarized significant factors influencing the optimal use of the tele-ICU from the nurses' perspective. |
| 32 | Telehealth, Telemedicine, and Related Technologic Platforms: Current Practice and Response to the COVID-19 Pandemic | Highlighting the differences between assistance with the aid of telemedicine and without such aid in the clinical practice of wound, ostomy, and continence (WOC) nurses | *Journal of Wound, Ostomy and Continence Nursing*; Mahoney (2020) | Telemedicine | Report | - The pandemic crisis has accelerated the need for health care to reinvent the delivery of care to patients. - Telemedicine technologies and principles have emerged as essential for WOC nurses to provide safe and effective care. |
| 33 | Exploring Readiness for Teleprecepting in Psychiatric Mental Health Nurse Practitioner Training | Supporting teleprecepting as a strategy to improve access to clinical training and as a resource for mental health nurses | *Journal of the American Psychiatric Nurses Association*; Johnson et al (2020) | Telemental health | Qualitative study | - Telemedicine during the pandemic can also expand nursing skills in the care of psychiatric patients. |
| 34 | Academic Nurse-Managed Community Clinics Transitioning to Telehealth: Case Report on the Rapid Response to COVID-19 | Case report on the rapid implementation of a telemedicine hub at a community clinic run by academic nurses in response to the national COVID-19 emergency | *JMIR Nursing*; Sutter et al (2020) | Telemedicine | Qualitative study | - The experiences of the clinics covered in this study, relating to the use of telemedicine during COVID-19, can be extended to other clinics. - The clinics were able to maintain most of the clinical service and health education functions by adapting to the new clinical tasks that arose during the pandemic. |
| 35 | Connecting With Families Through Virtual Perinatal Education During the COVID-19 Pandemic | Transformation of the birthing education program the Center for Perinatal Education and Lactation at New York University (NYU) Langone Hospitals into digital format to continue providing support and guidance to expectant families | *The American Journal of Maternal/Child Nursing*; Pasadino et al (2020) | Virtual format | Report | - The format allowed a quick response to the COVID-19 pandemic in support of expectant families, and thanks to live discussion webinar questions and answers, they were able to get in touch with them and alleviate anxiety and fear. |
| 36 | Primary-Care Registered Nurse Telehealth Policy Implications | Benefits of telemedicine through reimbursable primary care delivery systems | *Journal of Telemedicine and Telecare*; Watkins and Neubrander (2020) | Telemedicine and telenursing | N/A | N/A |
| 37 | How Telehealth Care Exploded due to COVID: What Nurse Researchers Need to Know | Editorial opinions regarding the changes, sanctioned by the COVID-19 pandemic, political changes, post–COVID-19 adjustments, and whether the volume of telemedicine will continue to be encouraged after the end of the emergency | Wiley Public Health Emergency Collection; Spaulding and Smith (2021) | Telehealth care | Editional opinions | - Future nursing researchers will need to know the political and administrative evolution of the large volumes of telemedicine projects that exploded during the years of the COVID-19 pandemic. |
| 38 | End-of-Life Care During COVID-19: Opportunities and Challenges for Community Nursing | Review of guidelines and experiences on care for patients dying from COVID-19 during the pandemic | *British Journal of Community Nursing*; Bowers et al (2020) | Teleassistenza | Review | - The challenge imposed by the pandemic is to find a way to accompany people, perhaps segregated at home, or perhaps unreachable, along the path of the end of life. |
| 39 | Impact of Technology on Community Nursing During the Pandemic | A critical analysis, reflection, and discussion on the uses and impacts that technology has had in community environments, particularly in nursing homes during the COVID-19 pandemic | *British Journal of Community Nursing*; Grindle (2021) | Virtual assessment platforms | Qualitative study | - The paper outlines the positives and critically reflects on the benefits of using audio- and videoconferencing when assessing patients and the beneficial impacts this has had on patients and the health community at large. - On the contrary, it also addresses the obstacles and threats encountered by doctors in using assessment software. |
| 40 | Adoption, Feasibility and Safety of a Family Medicine-Led Remote Monitoring Program for Patients With COVID-19: A Descriptive Study | Assessing the initial adoption, feasibility, and safety of a family drug–led remote monitoring program, COVIDCare @ Home, to manage the care of patients with COVID-19 in the community | National Library of Medicine; Agarwal et al (2021) | Telemonitoraggio | Quantitative study | - A team-based remote monitoring program can safely manage care for outpatients diagnosed with COVID-19. |

^a^N/A: not applicable.

**Table A3.** Summary of papers 41-60.

| Study number | Title | Aim | Journal/publisher; authors (year) | Telehealth/eHealth application | Data collection method | Key conclusion |
| --- | --- | --- | --- | --- | --- | --- |
| 41 | Virtual Health Care for Community Management of Patients With COVID-19 in Australia: Observational Cohort Study | Description of the implementation and first experience with virtual health care for community management of patients with COVID-19 | *Journal of Medical Internet Research;* Hutchings et al (2021) | Digital health | Quantitative study | - Health services implementing virtual health care should anticipate the challenges associated with rapid technology deployment and provide adequate support to address them, including strategies to support the use of health information technologies among consumers. |
| 42 | Effects of Telenursing Triage and Advice on Healthcare Costs and Resource Use | Presentation of an overview of current research, a description of the extent to which telenursing services are playing this role, identification of gaps in the literature, and proposed future research directions | *Studies in Health Technology and Informatics*; Gidora et al (2019) | Telemedicine | Report | - Greater evaluation of telenursing programs is needed to identify substantial savings. The health findings should be part of the research. |
| 43 | Telehealth and Telenursing Perception and Knowledge Among University Students of Nursing in Poland | Assessing the knowledge and attitude of nursing students toward telenursing | Mary Ann Liebert Inc; Glinkowski et al (2013) | Telemedicine and telenursing | Quantitative study | - The current generation of undergraduate nursing students appears to be well educated in medical informatics and the use of technology. They are better prepared and open to membership in the information society, including the practice of telemedicine. - Progress in university education has an influence on positive attitudes toward telenursing and can become a milestone in the development of eHealth in Poland. |
| 44 | The Influence of an eHealth Intervention for Adults With Type 2 Diabetes on the Patient-Nurse Relationship: A Qualitative Study | To explore how a program-based, guided self-determination (eGSD) eHealth intervention affects the patient-nurse relationship from the perspective of participating patients and nurses conducting the intervention | Wiley; Lie et al (2019) | Telenursing | Qualitative study | - EGSD influences the patient-nurse relationship by facilitating mutual understanding and flexibility. - Both patients and registered nurses (RNs) have recognized these findings as beneficial. However, in-person family consultations were expressed as an integral part of the patient-nurse relationship. |
| 45 | Conceptualizing Telehealth in Nursing Practice: Advancing a Conceptual Model to Fill a Virtual Gap | Conceptual model based on literature review showing the characteristics and relationships between dimensions of telemedicine practice to guide research and knowledge development in holistic person-centered care by nurses through telemedicine technologies | *Journal of Holistic Nursing*; Nagel and Penner (2015) | Telenursing | Review | N/A^a^ |
| 46 | Iranian Clinical Nurses' and Midwives' Attitudes and Awareness Towards Telenursing and Telehealth: A Cross-Sectional Study | Examining the attitude and awareness of Iranian clinical nurses and midwives toward telenursing and telemedicine, also known as eHealth | *Sultan Qaboos University Medical Journal*; Ranjbar et al (2021) | Telenursing and telemedicine | Quantitative study | - Most of the study respondents were aware of telenursing and telemedicine, and both nurses and midwives showed a positive attitude in using them, especially those with master’s degrees. - Implementing educational and infrastructure development programs can help speed up execution processes in these fields. |
| 47 | Challenges Posed by COVID-19 and Neurosurgical Nursing Strategies in Developing Countries | Overview of the challenges and strategies of neurosurgical nurses in the current COVID-19 pandemic environment in developing countries | *Surgical Neurology International*; Dhandapani and Dhandapani (2020) | Telehealth strategy and telenursing | Report | - Neurosurgical nursing care during the COVID-19 pandemic in developing countries requires transparent planning, implementation, and careful consideration of the various telemedicine strategies. |
| 48 | Effect of Remote Nursing Monitoring on Overweight in Women: Clinical Trial | Evaluating the effect of remote nursing monitoring on improving anthropometric measurements of overweight women | *Revista Latino-Americana de Enfermagem*; Palmeira et al (2019) | Remote nursing monitoring | Randomized clinical trial | - After being divided into 2 groups (one monitored by telemedicine mechanisms and the other in a conventional way), the women were analyzed after 3 months, showing a noticeable reduction in mean weight and mean BMI in the group followed with telemonitoring, useful also in reducing anthropometric measurements. |
| 49 | Nurses: Extending Care Through Telehealth | Highlighting the impact that telemedicine can have on clinical practice, expanding care capabilities, reducing costs and improper access, and resulting in a positive impact on both customers and suppliers | *IOS Press*; Allen et al (2015) | Telehealth | Report | - Telenursing goes beyond clinical support and has the potential to exponentially expand telehealth's services, normalizing it as a modality of care. - Nurses look to it to improve their ability to collaborate with remote patients, providing surgical care, maternal/pediatric care, and group education. |
| 50 | Performance of eHealth Data Sources in Local Influenza Surveillance: A 5-Year Open Cohort Study | To examine correlations between data from Google Flu Trends, computer-supported telenursing centers, health service websites, and influenza case rates during seasonal and pandemic flu outbreaks to carry out health surveillance against seasonal flu | *Journal of Medical Internet Research*; Timka et al (2014) | Telemedicine | Qualitative study | - Telemonitoring provides a possibility to improve surveillance systems. |
| 51 | Considerations for the Telehealth Systems of Tomorrow: An Analysis of Student Perceptions of Telehealth Technologies | To examine perceptions of telemedicine systems within a large sample of students | *JMIR Medical Education*; Bull et al (2016) | Telehealth application | Quantitative study | - By understanding the current barriers to adopting telemedicine in a cohort of students, we can not only better anticipate the future needs of this group but also incorporate those needs into the design of future telemedicine systems. |
| 52 | Perspectives of Nurses Toward Telehealth Efficacy and Quality of Health Care: Pilot Study | To examine the quality of the technology used by nurses | *JMIR Medical Education*; Bashir and Bastola (2018) | Telehealth technology | Pilot study | - The study highlighted how the telehealth process provides daily patient health monitoring, resulting in the benefits of immediate feedback for patients, family, and health care professionals, as well as the convenience of scheduling. |
| 53 | Analyzing Nursing Students’ Relation to Electronic Health and Technology as Individuals and Students and in Their Future Career (the eNursEd Study): Protocol for a Longitudinal Study | To provide nursing students with the basics to be able to go hand in hand with the development of telemedicine, given the improvement in services and an evolution in hospital technology in recent decades | *Journal of Medical Internet Research*; Anderberg (2019) | Telehealth | Longitudinal study and qualitative substudy | - The knowledge from this study will be used to compare different nursing programs and students' knowledge and relationships with technology and eHealth. This will provide university centers with an opportunity to improve the course of study in health informatics and technology. |
| 54 | Academic Nurse-Managed Community Clinics Transitioning to Telehealth: Case Report on the Rapid Response to COVID-19 | Study carried out by George Mason University’s Mason and Partners clinics to evaluate telemedicine used in COVID-19 times, also identifying the success factors and challenges associated with the transition to telemedicine | *Journal of Medical Internet Research*; Sutter et al (2020) | Telehealth | Qualitative review | - The changes resulting from the pandemic have led to sustainable procedures, and these changes will have a long-term impact on health care delivery and training. |
| 55 | The Associations of Electronic Health Record Usability and User Age With Stress and Cognitive Failures Among Finnish Registered Nurses: Cross-Sectional Study | Examining the use of electronic devices by nurses with stress related to the computer systems used based on age | *Journal of Medical Internet Research*; Kaihlanen et al (2020) | Questionari riguardi le cartelle sanitarie elettroniche | Quantitative study | - Stress related to the use of obsolete computer systems affects elderly and young nurses. Younger ones are far more affected as they find these systems difficult to use, increasing the possible risk of errors and stress. |
| 56 | Perspectives of Nurses and Doulas on the Use of Information and Communication Technology in Intercultural Pediatric Care: Qualitative Pilot Study | To investigate the role of information and communication technology (ICT) in managing communication challenges related to language problems and cultural differences in encounters with migrant mothers from the perspective of Swedish pediatric nurses | *Journal of Medical Internet Research*; Lindström et al (2020) | ICT | Qualitative pilot study | - The results of this study suggest that ICT can be a link between health care professionals and migrants. - The advantages and disadvantages of translation tools should be discussed to ensure that communication is of quality in health interactions. - This study also suggests the development of targeted multimodal digital support, including graphics and video resources, for pediatric care services. |
| 57 | Development and Implementation of a Nurse-Led Model of Care Coordination to Provide Health-Sector Continuity of Care for People With Multimorbidity: Protocol for a Mixed Methods Study | Implementing a nurse-led care coordination model from an outpatient setting to provide continuity of secondary and primary care for people with comorbidities | *Journal of Medical Internet Research*; Davis et al (2019) | Telehealth | Systematic pilot study | - The findings and experiences of nursing clinical and patient care with the nurse-led care coordination model will provide a model for improving continuity of care between secondary and primary health systems. - The model can provide a future path for the implementation of nurse-led services both nationally and internationally. |
| 58 | The Effects of Telenurse Care (by Mobile) on the Patients Prior to Suicide Attempt with Anxiety & Depression Disorders | To examine telephone contact by psychiatrists and nurses before a suicide attempt due to anxiety and depression in a Tehran outpatient hospital | Elsevier; Kavari (2014) | Short messaging service (SMS), mobile phone, questionnaire | Qualitative study | - Knowledge of suicidal behavior patterns is necessary for suicide prevention programs. Many studies have shown that those attempting suicide had contact with doctors prior to the suicide attempt. Therefore, it is suggested that nurses and other medical groups use mobile distance learning in suicide programs. |
| 59 | Telephone Nurses' Communication and Response to Callers' Concern: A Mixed Methods Study | To describe the communication between the telephone nursing service and patients; to evaluate the direct response of telephone nurses to the expressions of concern of the callers | Elsevier; Ernesäter et al (2015) | Telephone | Mixed methods study | - The reluctance of telephone nurses to use open-ended questions and follow up on caller understanding could be a threat to concordance and a potential threat to patient safety. |
| 60 | Personalized Care Management for Persons With Parkinson's Disease: A Telenursing Solution | To study how telenursing is useful to prevent Parkinson symptoms that lead to a progressive limitation of motor functions with the risk of falls and injuries | Elsevier; Mancini (2020) | Telephone | Quantitative study | - During a 3-month intervention period that included 13 telephone contacts, the patients reported a notable reduction in the number of falls, from 99 falls every 3 months to 3 falls every 3 months, and a reduction in nonmotor symptoms. - The main working mechanism was presumably rather indirect and mediated through the alleviation of anxiety, achieved thanks to the personalized information and problem-solving strategies provided by the PDNS |

^a^N/A: not applicable.
